# Outcomes and complications of metallic vs. bioabsorbable screw fixation for displaced medial epicondylar fractures in pediatric and adolescent patients

**DOI:** 10.1007/s00068-026-03278-2

**Published:** 2026-07-29

**Authors:** Tobias Jhala, Simon Scherer, Michael Esser, Justus Lieber, Jörg Fuchs, Markus Dietzel

**Affiliations:** 1 Department of Pediatric Surgery and Pediatric Urology, University Children’s Hospital Tuebingen, Hoppe-Seyler-Str 3, 72076 Tuebingen, Germany; 2 Department of Radiology, Diagnostic and Interventional Radiology, University of Tuebingen, Tuebingen, Germany

**Keywords:** Pediatric, Dislocation, Elbow fracture, Bioabsorbable screw, Comparison

## Abstract

**Purpose:**

The use of bioabsorbable screws in orthopedics and trauma surgery is increasing. The major advantage of not having to remove the implant is of particular interest in pediatric surgery. Due to drawbacks, most notably implant fracture, bioabsorbable screw osteosynthesis has not yet surpassed the gold standard of metallic screw osteosynthesis. This single-centre study compares metallic screw and bioabsorbable screw osteosynthesis of medial humeral epicondyle fractures in children and adolescents over a two-year period.

**Materials and methods:**

Retrospective analysis of surgically treated (screw fixation with either bioabsorbable magnesium-based or metallic screw) patients with a medial humeral epicondyle fracture between 2020 and 2022 at the author’s institution.

**Results:**

Seventeen patients with medial epicondyle fractures underwent surgical treatment using either bioabsorbable screws (*n* = 8) or metallic screws (*n* = 9). Baseline characteristics, surgical approach, and postoperative care were similar between groups. Two complications (Clavien-Dindo I) occurred in the bioabsorbable group due to implant failure (screw breakage), though without clinical sequelae. These implant failures led to a significantly increased number of postoperative radiographs in the bioabsorbable group (median 4.8 vs. 2.5; *p* = 0.014). Functional outcomes (LOM, MEPI) and follow-up durations were comparable between groups.

**Conclusion:**

Bioabsorbable screws appear to be a promising alternative to conventional metallic fixation in the treatment of pediatric medial epicondyle fractures. Although screw breakage—and the associated increase in radiographic surveillance—remains a notable concern, advances in biomaterial technology may help reduce this complication in the future.

## Introduction

The utilisation of bioabsorbable screws (BAS) has been increasing for three decades. Constant developments and improvements in this field have rendered them not only of interest to orthopedic and maxillofacial surgery, where screws and osteosynthesis must withstand less force, but also to trauma surgery. The most significant benefit of bioabsorbable screws is that they do not require removal. This is a particularly salient benefit in pediatric surgery, where implant removal is almost always performed under general anaesthesia. Nevertheless, concerns regarding the potential for implant fracture have impeded the widespread adoption of bioabsorbable screws, which thus far has not been able to surpass the established metallic screw (MS) osteosynthesis as the gold standard in terms of complication rates [1]. One fracture type in which bioabsorbable screws are utilized are epicondylar avulsion fractures in children. Epicondylar avulsion injuries are the third most prevalent elbow fracture type in children [2]. The medial epicondyle is affected in the vast majority of cases (99%). A notable aspect of this injury is its association with elbow dislocation, which occurs in up to 60% of cases [3, 4]. In recent decades, while the optimal treatment strategy is still debated, a marked tendency towards surgical intervention and early postoperative mobilisation has been observed in the treatment of medial epicondylar fractures in children. In such cases, screw osteosynthesis is considered the optimal treatment modality [5].

However, recent high-level randomized evidence has questioned this treatment paradigm. Large multicentre trials have demonstrated that non-operative treatment may achieve comparable functional outcomes to surgical fixation at 12 months, despite differences in radiographic healing and complication profiles [6, 7].

Importantly, these findings apply to heterogeneous fracture populations. Surgical fixation remains indicated in selected cases, particularly in the presence of joint instability, incarcerated fragments, or neurological compromise, where conservative management is not considered appropriate [6, 7].

Against this background, the aim of this study was to compare functional outcomes and the frequency of postoperative radiographic follow-up in children with medial humeral epicondyle fractures treated with either bioabsorbable or metallic screw fixation.

## Materials, methods and ethical considerations

### Patients and ethical considerations

All patients below 16 years of age with a surgically treated (screw osteosynthesis) fracture of the medial humeral epicondyle treated at the author’s institution between 1/2020 and 12/2022 were retrospectively analysed. Demographic characteristics, clinical background, indication for surgery, intraoperative findings, frequency of radiologic follow-up and postoperative outcomes were collected from hospital records.

Open reduction and internal fixation (ORIF) was indicated in cases involving incarcerated fragments, significant displacement, joint instability (defined as spontaneous dislocation of the elbow joint during gentle movement under fluoroscopy) or associated neurological symptoms [5, 8]. Surgical treatment consisted of open reduction and internal screw fixation. The screw used, bioabsorbable (MAGNEZIX - Syntellix AG, Hannover, Germany) or metallic screw (titanium cannulated screw – DePuy Synthes, West Chester, USA), was chosen upon surgeonsˋ preference. The MAGNEZIX screw is now no longer commercially available.

In all cases, the screws were applied strictly according to the manufacturer’s surgical technique guidelines.

Postoperative management consisted of early mobilization using an orthosis. All patients were followed up in the outpatient clinic, where elbow range of motion (ROM) was assessed using the neutral-zero method. A normal ROM was defined as 5–0–145 degrees for extension and flexion, along with a physiological cubitus valgus of 5 degrees. Outcomes were classified as excellent when patients were pain-free and showed no limitations in elbow extension or flexion during clinical examination. Results were considered fair in patients with a mild loss of motion (LOM) of 10° or less, and moderate in those with an LOM greater than 10°. For standardized functional evaluation, the Mayo Elbow Performance Index (MEPI) was used [9].

### Statistics

Statistical analysis of the different treatment groups (bioabsorbable screw and metallic screw) was assessed using computing Student’s t-test. All tests were performed using Microsoft Excel (Microsoft Corp., Redmond, WA, USA). All *p*-values below 0.05 were considered statistically significant. Given the small sample size, statistical comparisons were considered exploratory. *p*-values were reported for descriptive purposes only and were not used to draw definitive conclusions.

Ethical considerations: The study was conducted following the ethical principles outlined in the Declaration of Helsinki (DoH) and the guidelines of the International Council for Harmonization Good Clinical Practice (ICH GCP). The study was approved by the local ethical committee (project number 629/2019BO2).

## Results

A total of 17 patients with medial epicondyle fractures treated surgically were included. Eight patients received bioabsorbable screws, and nine were treated with metallic screws. The median age was similar between the groups (BAS: 12.4 years, SD 1.2; MS: 11.3 years, SD 2.3). All injuries occurred during recreational activities. Two patients in each group sustained associated elbow dislocations.

All patients underwent open reduction and screw osteosynthesis. The choice of implant was based upon surgeon’s preference. Metallic screws used had diameters of 3.5 or 4 mm, while bioabsorbable screws measured 3.2 mm in diameter. No intraoperative or perioperative complications were observed.

**Fig. 1:**
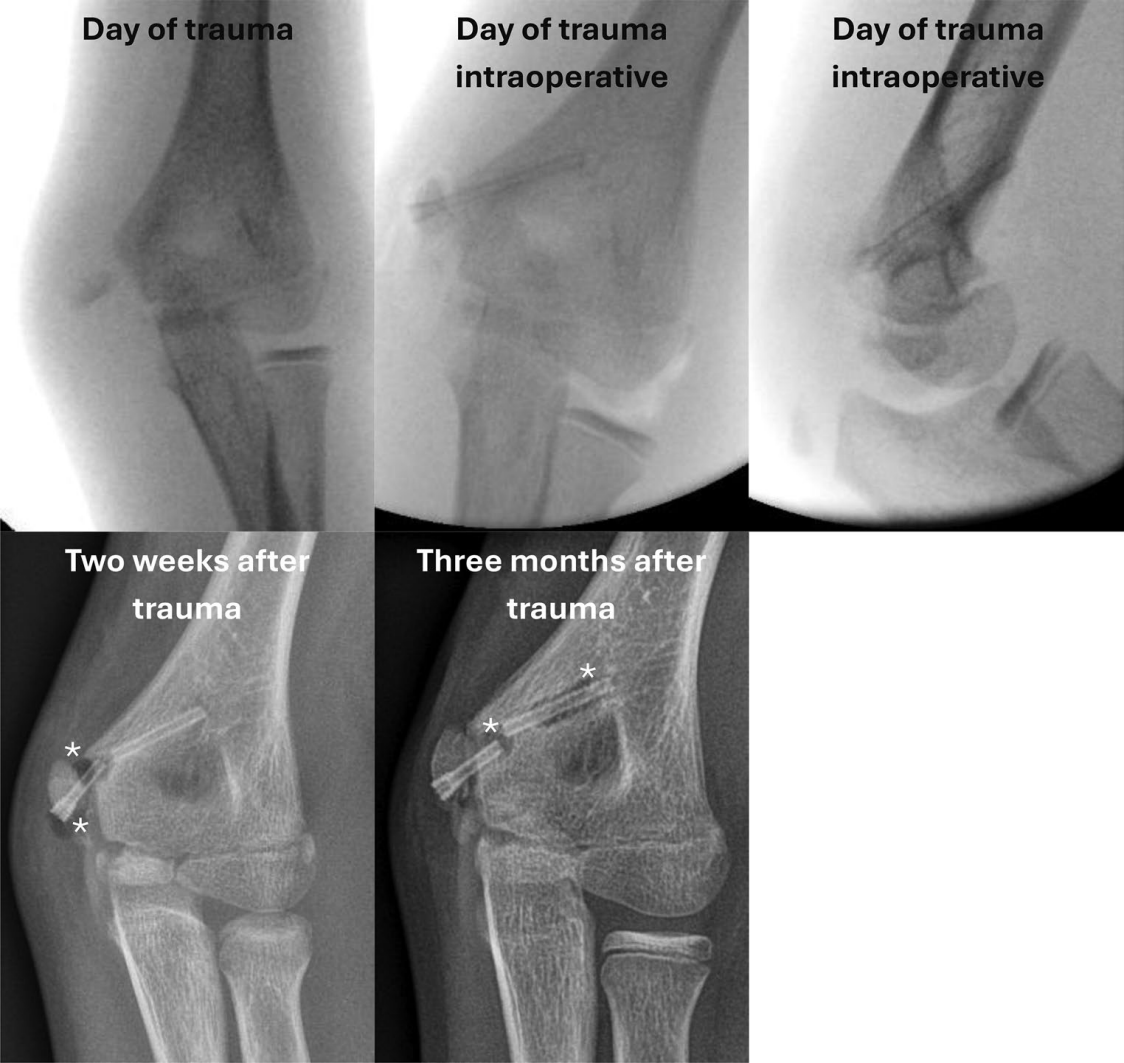
Illustrative case of early screw breakage one week after surgery but subsequent uneventful healing. Resorption zones with physiologiscal gas bubbles in the bone and soft tissue surrounding the screw is marked with asterixis

Postoperative management, including duration of immobilization in a cast and orthosis, was similar between the groups. Two complications (Clavien-Dindo Grade I) occurred, both in the BAS group, involving implant failure (screw breakage) as seen in Fig. 1.

Although not statistically significant (*p* = 0.08), both failures occurred within two weeks postoperatively. These cases did not result in clinical sequelae but required prolonged cast immobilization (four weeks instead of two) and more frequent radiologic follow-up to monitor for potential fragment displacement. Consequently, the BAS group underwent a significantly higher number of postoperative radiographs (median 4.8, SD 1.7) compared to the MS group (median 2.5, SD 1.6; *p* = 0.014). Patients with bioabsorbable screws and screw breakage underwent six to seven radiographs (anteroposterior and lateral views), whereas those with bioabsorbable screws without breakage underwent only two to four radiographs. Uneventful screw removal in the MS group was performed after a median of 16.6 weeks (SD 4.9). Return to sport was allowed immediately after the end of mobilization in the orthosis.

Mean follow-up after surgery duration was comparable (BAS: 2.5 months, SD 2.2; TS: 3.7 months, SD 1.4). Functional outcomes measured by the Mayo Elbow Performance Index (MEPI) were similar between groups: in the BAS group, two patients achieved excellent and five good outcomes; in the MS group, five were rated excellent, two good, and two fair. Results are detailed in Table 1.

**Table 1:** Patient characteristics and outcomes of both treatment groups

	Bioabsorbable screws	Metallic screws
Patients (n)	8	9
Mean age (years) [SD]	12.4 [1.2]	11.3 [2.3]
Associated elbow joint dislocation (n)	2	2
Mean operative time (minutes) [SD]	53.2 [14.3]	58.9 [23.3]
Cumulative intraoperative fluoroscopy dose (mGy) [SD]	0.58 [0.2]	0.57 [0.3]
Mean time of cast immobilisation (weeks) [SD]	2.4 [1.1]	2.6 [1.6]
Mean time of mobilization with orthosis (weeks) [SD]	3.3 [1.1]	2.7 [1.6]
Complications (n)	2	0
Mean number of radiographs (n) [SD]	4.8 [1.7]	2.5 [1.6]
Mean time to screw removal (weeks) [SD]	-	16.6 [4.9]
Mean follow-up (months) [SD]	2.5 [2.2]	3.7 [1.4]
LOM (n) [none I mild I moderate]	3 I 5 I 0	5 I 4 I 0
MEPI (n) [excellent I good I fair I poor]	2 I 6 I 0 I 0	5 I 2 I 2 I 0

## Discussion

Medial epicondyle fractures of the distal humerus are among the most common elbow injuries in the pediatric population [2]. While the indication if surgery is needed are still discussed, open reduction and internal fixation (ORIF) is generally indicated in cases involving incarcerated fragments, significant displacement, joint instability or associated neurological symptoms [5, 8].

Although recent randomized controlled trials have shown that non-operative treatment can achieve comparable functional outcomes to surgical fixation in many displaced medial epicondyle fractures, surgical management remains indicated in cases with clear mechanical or neurological indications. In the present study, all included patients met established clinical criteria for operative treatment, including joint instability, incarcerated fragments, or neurological symptoms [6, 7].

Common fixation techniques include the use of metallic screws and Kirschner wires (K-wires) [5, 10]. Compression screws, when fragment size permits, offer superior biomechanical stability compared to K-wires, thereby facilitating early mobilization and reducing the risk of postoperative elbow stiffness. Cast immobilization only being necessary for postoperative analgetic effect [5]. However, metallic implants may be associated with local soft tissue irritation or cosmetic disturbance (screw prominence) and often necessitate a second procedure for removal, increasing the overall healthcare burden [5, 11, 12].

Bioabsorbable materials have emerged as a potential alternative in fracture fixation to address these limitations. Their use has been documented in various orthopedic, neurosurgical, and maxillofacial applications. In this study, outcomes following fixation with bioabsorbable screws (MAGNEZIX - Syntellix AG) were compared to those with metallic screws in the setting of pediatric medial epicondyle fractures.

Seventeen patients were included, with comparable baseline characteristics and standardized perioperative management. Nine patients were treated with metallic screws, eight with bioabsorbable screws. The median age of patients aligned with the reported peak incidence of medial epicondyle fractures, typically observed between 10 and 12 years of age [13].

While no intraoperative complications occurred in either group, two cases of screw breakage were observed in the bioabsorbable screw (BAS) group within the first two postoperative weeks. This resulted in a statistically significant increase in radiographic follow-up in this cohort (*p* = 0.014). Early implant failure therefore represents a relevant finding requiring careful interpretation. The very early timing of failure suggests that this is unlikely to be primarily driven by material degradation, but rather reflects a combination of mechanical factors including implant design, screw diameter, construct stability, and early functional loading conditions. However, a possible cofounder was the smaller screw diameter of the bioabsorbable screws compared to the metallic screws. Screw breakage, though infrequent, has been previously reported in the literature [10, 14].

A known risk factor is small screw diameter, particularly those below 2 mm [14]. The location of osteosynthesis does not appear to significantly influence this complication, as successful applications have been documented even in weight-bearing bones [15].

Therefore, the observed screw breakages raise the question of whether the material itself, specifically magnesium-based screws (in our cohort MAGNEZINX [MgYREZr]), contributed to the mechanical failure. Although magnesium alloys are considered promising due to their biocompatibility and osteoconductive properties, it is well known that they can reabsorb relatively rapidly under physiological conditions, potentially leading to premature loss of mechanical integrity [16].

Another characteristic of magnesium-based implants is peri-implant gas accumulation, which occurs as a result of the corrosion process. Recent evidence suggests that peri-implant gas accumulation is a common radiographic finding around magnesium implants and may occur both intraosseously and in adjacent soft tissues. In our cohort, gas accumulation was observed radiographically around magnesium screws but was not associated with clinical symptoms, impaired fracture healing, or revision surgery. Nevertheless, this phenomenon should be considered when interpreting postoperative imaging of biodegradable magnesium implants [17].

Recent developments have focused on creation of new alloys and optimizing available magnesium alloys with reduced corrosion rates and improved mechanical strength. Preliminary preclinical data on alloys such as MgCaZn suggest superior structural stability compared to pure magnesium implants [18].

Alternatively, polymer-based bioresorbable materials such as poly-L-lactic acid (PLLA) have been proposed. While they offer slower degradation and more consistent mechanical properties over several weeks, they show lower primary stability possibly making them more prone to early implant failure [10, 15, 19, 20].

In addition to material-related factors, biomechanical aspects must also be considered. Early functional mobilization, increasingly advocated in postoperative protocols, could further intensify mechanical loading during the vulnerable early healing phase. This raises the question of whether temporary unloading, for example via an additional bioresorbable anchor or a protective orthosis, might have helped mitigate stress during this period [21].

Notably, in the present study and in previously reported cases, screw breakage did not necessitate revision surgery or alter long-term functional outcomes [10].

However, absence of revision surgery should not be interpreted as absence of clinical relevance. Implant failure may still influence postoperative management, increase imaging requirements, and generate uncertainty in early follow-up, even in the absence of functional impairment.

Functional results, as measured by MEPI and range-of-motion assessments, were comparable between the BAS and metallic groups. These findings suggest that despite the increased imaging burden, bioabsorbable screws do not compromise clinical outcomes [5, 10, 11, 22]. Furthermore, the elimination of hardware removal and a reduced incidence of hardware prominence present important advantages [19]. Moreover, BAS become more widely utilized, surgeons and radiologists have become more familiar with postoperative imaging variants in the reabsorption process, it’s likely that the noted increased need for imaging will decrease again [1].

Nevertheless, certain limitations must be acknowledged, including the small sample size and the retrospective design, which may introduce selection bias and limit generalizability. In addition to material-related properties, implant diameter and surgical technique are likely to play a crucial role in the mechanical stability of fixation. In the present study, the use of smaller diameter bioabsorbable screws may have contributed to reduced mechanical stability and could represent a contributing factor to early implant failure. Additionally, while no adverse foreign body reactions were observed during the study period, longer-term follow-up is required to assess this risk more comprehensively.

## Conclusion

Bioabsorbable screws appear to be a promising alternative to conventional metallic fixation in the treatment of pediatric medial epicondyle fractures. The greatest advantage of bioabsorbable screws is that no metal removal—and therefore no second anaesthesia—is necessary. Although screw breakage—and the associated increase in radiographic surveillance—remains a notable concern, advances in biomaterial technology may help reduce this complication in the future. To better define the clinical utility of bioabsorbable fixation, further high-quality prospective studies with larger patient cohorts are needed to assess long-term safety, cost-effectiveness, and functional outcomes in pediatric trauma populations.

## Data Availability

The datasets generated and analyzed during the current study are not publicly available due to institutional data protection regulations and the inclusion of potentially identifiable patient information. De-identified data may be made available from the corresponding author on reasonable request and with permission of the local ethics committee (project number 629/2019BO2), in accordance with applicable data protection laws.

## Abbreviations

ROM: Range of motion
LOM: Loss of motion
MEPI: Mayo Elbow Performance Index
BAS: Bioabsorbable screw
MS: Metallic screw

## Declarations

### Submission declaration

This work has not been published previously and is not under consideration for publication elsewhere. This publication is approved by all authors.
